# Functional Contribution of the Transcription Factor ATF4 to the Pathogenesis of Amyotrophic Lateral Sclerosis

**DOI:** 10.1371/journal.pone.0066672

**Published:** 2013-07-18

**Authors:** Soledad Matus, Estefanía Lopez, Vicente Valenzuela, Melissa Nassif, Claudio Hetz

**Affiliations:** 1 Neurounion Biomedical Foundation, Santiago, Chile; 2 Biomedical Neuroscience Institute, Faculty of Medicine, University of Chile, Santiago, Chile; 3 Center for Molecular Studies of the Cell, Program of Cellular and Molecular Biology, Institute of Biomedical Sciences, University of Chile, Santiago, Chile; 4 Department of Immunology and Infectious Diseases, Harvard School of Public Health, Boston, Massachusetts, United States of America; Baylor College of Medicine, Jiao Tong University School of Medicine, United States of America

## Abstract

Endoplasmic reticulum (ER) stress represents an early pathological event in amyotrophic lateral sclerosis (ALS). ATF4 is a key ER stress transcription factor that plays a role in both adaptation to stress and the activation of apoptosis. Here we investigated the contribution of ATF4 to ALS. ATF4 deficiency reduced the rate of birth of SOD1^G86R^ transgenic mice. The fraction of ATF4^−/−^-SOD1^G85R^ transgenic mice that were born are more resistant to develop ALS, leading to delayed disease onset and prolonged life span. ATF4 deficiency completely attenuated the induction of pro-apoptotic genes, including BIM and CHOP, and also led to quantitative changes in the ER protein homeostasis network. Unexpectedly, ATF4 deficiency enhanced mutant SOD1 aggregation at the end stage of the disease. Studies in the motoneuron cell line NSC34 demonstrated that knocking down ATF4 enhances mutant SOD1 aggregation possibly due to alteration in the redox status of the cell. Our results support a functional role of ATF4 in ALS, offering a novel target for disease intervention.

## Introduction

Amyotrophic lateral sclerosis (ALS) is a progressive and deadly adult-onset motoneuron disease characterized by muscle weakness, spasticity, atrophy, paralysis and premature death. The pathological hallmark of ALS is the selective degeneration of motoneurons in the spinal ventral horn, most brainstem nuclei and cerebral cortex [1], [2]. The majority of ALS patients lacks a defined genetic component, and is considered sporadic, while approximately 10% of cases are familial (fALS) [1]. Many disease-causative genes have been identified; including hexanucleotide-repeat expansion in the intronic region of *C9orf72*, superoxide dismutase-1 (*SOD1*), *TARDBP*, *FUS*, and many others [1]. Overexpression of fALS-linked SOD1 mutants in transgenic mice recapitulates essential features of the human pathology, provoking age-dependent protein aggregation, paralysis, motoneuron degeneration and muscle atrophy [2], [3]. The primary mechanisms contributing to the progressive motoneuron loss in ALS remains controversial, and multiple perturbations of cellular function/processes have been uncovered in ALS-affected motoneuron and glial cells [3]. Accumulating evidence highlights a functional involvement of endoplasmic reticulum (ER) stress in both sALS and fALS pathogenesis, representing an early event in the pathology [4], [5], [6].

ER stress is triggered by a number of conditions that interfere with the oxidative-protein folding process at the ER lumen, leading to accumulation of abnormally folded proteins [7]. ER stress engages the unfolded protein response (UPR), an integrated signal transduction pathway that increases the protein folding capacity and quality control mechanisms of the ER, mitigating the load of unfolded proteins [8]. Conversely, chronic ER stress results in apoptosis of irreversibly damaged cells through diverse complementary mechanisms [9]. The UPR is activated by three main stress sensors, including PKR-like ER kinase (PERK), Inositol-requiring transmembrane kinase/endonuclease (IRE1), and activating transcription factor 6 (ATF6). Upon activation PERK reduces protein translation into the ER by phosphorylating the eukaryotic initiation factor 2 alpha (eIF2α), which contributes to attenuate the misfolded protein overload [10]. In addition, phosphorylation of eIF2α allows the expression of activating transcription factor 4 (ATF4), a key factor that upregulates UPR genes that function in amino acid and redox metabolism, autophagy, protein folding, and apoptosis [7], [11], [12], [13]. PERK has dual signaling outputs toward promoting cell survival or the induction of cell death by integrating information about the intensity and kinetic of exposure to ER stress. In the control of apoptosis, ATF4 drives the expression of the transcription factor GADD153/CHOP, modulating the levels of multiple pro-apoptotic components including BIM and PUMA (see examples in [14], [15], [16]), and could also induce BIM expression through the microRNA pathway as we recently described [17]. Sustained PERK signaling also contributes to apoptosis by enhancing oxidative stress and by resuming protein synthesis after prolonged ER stress [18], [19], [20]. Although PERK deficiency enhances the susceptibility of cells to apoptosis in mouse embryonic fibroblasts [11], its expression in neurons has been shown to operate as a central regulator of oxidative stress genes affecting cell survival during hypoxia [21]. In contrast, another study suggested that CHOP could also have a neuroprotective activity against hypoxia [22]. PERK/ATF4 also enhances the expression of the anti-apoptotic protein GRINA, modulating ER calcium homeostasis [23]. In addition to ER stress, the eIF2α/ATF4 pathway can be engaged by other stimuli involving kinases stimulated by viral infections, metabolic alterations, among other factors, suggesting a broad function of the eIF2α/ATF4 pathway as a stress integrator. IRE1 controls the expression of the transcriptional factor X-Box-binding protein 1 (XBP1) through an unconventional splicing process, whereas processing of ATF6 at the Golgi releases the cytosolic domain that acts as a transcription factor [8]. Both XBP1 and ATF6 control a subset of UPR-target genes related to protein folding and quality control mechanisms. Overall, UPR signaling responses modulate the expression of a large spectrum of partially overlapping target genes to recover ER homeostasis or to trigger cell death programs depending on the nature of the stress stimuli [9].

Different groups have reported the activation of the UPR in human post-mortem samples derived from ALS patients [24], [25], including XBP1 and ATF4 expression as we reported [26]. The occurrence of ER stress has been also recapitulated in most cellular and animal models of fALS (reviewed in [4], [5]). Remarkably, a systematic gene-expression profile study in mutant SOD1 transgenic mice indicates that only affected motoneurons are selectively prone to undergo chronic ER stress, occurring before the earliest denervation in asymptomatic animals [27]. The possible effects of decreasing ER stress levels in ALS were tested using pharmacological approaches. Treatment of mutant SOD1 mice with salubrinal, a small molecule that selectively induces eIF2α phosphorylation, significantly protected against disease progression in a small cohort of animals [27]. Consistent with this report, *perk* haplo-insufficiency (PERK^+/−^ mice) exacerbated the severity of experimental ALS, associated with enhanced neuronal loss and mutant SOD1 aggregation [28]. Surprisingly, the loss of one *perk* allele was not sufficient to attenuate the induction of ATF4 in early symptomatic mice, reflected in unaltered induction of CHOP and BiP in *Perk^+/−^*-mutant SOD1 mice [28]. Thus, the protective effects of targeting PERK in this study are possibly attributed to a mild inhibition of protein translation. In contrast, we showed that XBP1 deficiency protects against the development of ALS [26]. Similarly, targeting ASK1, a downstream signaling component of IRE1 [29], or ablating the expression of BIM or PUMA delays ALS [30], [31]. These studies illustrate the complex nature of the UPR, where the functional impact of the pathway to ALS may actually depend on the specific outputs regulated by particular UPR signaling modules [9]. Here we investigated the specific contribution of ATF4 to ALS pathogenesis. Our results uncovered a complex scenario where ATF4 deficient animals were significantly resistant to develop ALS pathology. The protective effects of ATF4 deficiency were related to the down-regulation of the pro-apoptotic factors including CHOP and BIM, and altered expression of key ER foldases. Our results indicate a functional contribution of ATF4 in ALS pathogenesis.

## Materials and Methods

### Ethics Statement

The animal care and all animal experiments were performed according to procedures approved by “Guide for the Care and Use of Laboratory Animals” (Commission on Life Sciences. National Research Council. National Academy Press 1996) and approved by the Bioethical Committee of the Faculty of Medicine, University of Chile (protocol number CBA0399 FMUCH).

### Materials and Plasmids

Tunicamycin (Tm), pepstatin, E64D, and puromycin were purchased from Sigma (St Louis, MO, USA). Cell media and antibiotics were obtained from Life Technologies (Maryland, USA). Fetal calf serum was obtained from Atlanta Biologicals (Norcross, GO, USA). DAPI was purchased from Molecular Probes (Eugene, OR, USA). All transfections were performed using the Effectene reagent (Qiagen, Germantown, MD, USA). DNA was purified with Qiagen kits.

The human SOD1-EGFP expression vectors (the wild-type and the G85R mutant) were provided by Dr. Julie Atkin and described in [32]. In brief, primers were designed to introduce a *Sal*I site to allow subcloning SOD1 mutants into pEGFP-N1 (Clontech, Palo Alto, CA) and to remove the SOD1 translation stop codon. Mutants were generated via site directed mutagenesis of the SOD1^WT^ template using the Quick Change kit (Stratagene, La Jolla, CA). The plasmids to express HyPer-ER_lum_ and ERO1Lα-mCherry were described before [33]. The HyPer-ER_lum_ consists in a modified fluorescent protein that acts as a high sensitive H_2_O_2_ biosensor developed by Belousov *et al*. [34]. In the presence of H_2_O_2,_ the excitation peak at 420 nm of HyPer-ER_lum_ is reduced proportionally to the increased in the peak at 500 nm. The construct was modified by Enyedi *et al.* making this sensor target to the lumen of the ER [33] and allows the measure of H_2_O_2_ levels at the ER in living cells. The pcDNA3.1/V5 plasmids to express PDI and ERp57 were kindly provided by Dr. Neill Bullheid (University of Glasgow, UK).

### Knockdown of UPR components

We generated stable motoneuron cell lines with reduced levels of ATF4 using methods previously described [26] by targeting their respective mRNAs with shRNAs using the lentiviral expression vector pLKO.1 and puromycin selection. As control, empty vector or shRNA against the *luciferase* gene were employed. Constructs were generated by The Broad Institute (Boston, USA), based on different criteria for shRNA design (see http://www.broad.mit.edu/genome_bio/trc/rnai.html) [35]. Targeting sequence identified for mouse ATF4 was 5′-CCGG-GCGAGTGTAAGGAGCTAGAAA-CTCGAG-TTTCTAGCTCCTTACACTCGC-TTTTTG-3′.

### Assays for mutant SOD1 aggregation and detection of intracellular inclusions

We developed assays using the transient expression of human SOD1^WT^ and the mutant and SOD1^G85R^ as EGFP fusion proteins [26]. These constructs were employed to visualize and quantify the formation of intracellular SOD1 inclusions in living cells by fluorescent confocal microscopy. SOD1-containing aggregates were visualized in total cell extracts prepared in 1% Triton-X-100 in PBS buffer containing protease inhibitors followed by sonication and Western blot analysis. For samples treated with DTT, protein extracts were incubated with 100 mM DTT at room temperature for 10 min.

### Fluorescence measurements

H_2_O_2_ levels were assessed using a 40×/1.4 NA oil immersion objective in an IX-81 inverted microscope for fluorescence measurements (DSU, Olympus), equipped with a 150-W xenon lamp (Olympus MT-20). For ratiometric measurement, the HyPerERlum sensor was excited at 490/420 nm wavelengths and the fluorescence was filtered at 510 nm was collected and recorded at 0.2 Hz using a CCD-based imaging system (Olympus DSU). Coverslips were placed in a chamber and mounted on the microscope. Cells were incubated in 0.5 ml fresh extracellular medium containing 145 mM NaCl, 5 mM KCl, 1 mM MgCl2, 2 mM CaCl2, 10 mM HEPES, 10 mM glucose, pH 7.4 kept at 23°C. After acquisition of the baseline measurement, DTT and H_2_O_2_ were added to a final concentration of 10 mM and 1 mM, respectively, to ensure the functionality of the sensor. The CellR software (Olympus) software was used for data acquisition. Images were acquired every 15 s for a period of 300 seconds. The 490/420 nm fluorescence excitation ratio of HyPer-ER_lum_ was calculated after background fluorescence subtraction. Mean fluorescence intensities over individual cells were calculated from 2 min recordings. For time-resolved measurements of fluorescence, background subtracted recordings were averaged and plotted against time. Endogenous peroxides were measured by incubating control cells and treated with 2.5 µM tunicamycin for 4 h in medium containing 5 µM of 5-(and-6)-chloromethyl-2′, 7′ dichlorodihydrofluoresceine diacetate (H2DCFDA, Molecular Invitrogen) at 37°C in a 5% CO_2_ incubator followed by FACscan analysis.

### Animal Experimentation

Animals were maintained in a quiet, ventilated and temperature controlled room (23°C) and monitored daily. Mice were housed in a polystyrene solid bottom plastic cage fitted with a functioning filter top. Animals were fed with LabDiet pellets and had available drinking water *ad libitum*. Animals were maintained with a standard 12 h light cycle. For animal euthanasia, animals received intraperitoneal injection of anesthesia (100 mg/kg ketamine plus 10 mg/kg xylazine). ATF4 deficient mice were previously described [36], [37]. ATF4 deficient animals were obtained from Dr. Laurie Glimcher laboratory (Harvard School of Public Health, Boston MA, USA) where they were backcrossed to C57BL/6 pure background. We employed as an ALS model a SOD1^G86R^ transgenic strain (the equivalent of human SOD1^G85R^) originally generated in the FVB/N strain (strain FVB-Tg(Sod1-G86R)M1Jwg/J, The Jackson Laboratory) [38] in which the expression of the SOD1 mutant gene is driven by the endogenous SOD1 promoter. Mutant SOD1 mice were backcrossed to C57BL/6 for more than six generations. ATF4^+/−^ mice were then crossed with ATF4^+/−^/SOD1^G86R^ transgenic mice to generate experimental animals. We obtained 8 ATF4^−/−^/SOD1^G86R^ mice (4 males and 4 females) from 463 animals obtained (see table 1).

**Table 1:** Proportion of mice obtained in the generation of ATF4^+/+^-SOD1^G86R^ and ATF4^−/−^-SOD1^G86R^ mice.

	Number of animals generated:		
	Non Tg	SOD1^G86R^	Viable SOD1^G86R^
			(%)
ATF4^+/+^	39	36	48
ATF4^+/−^	231	134	36,7
ATF4^−/−^	15	8	34,7
Total Animals	463		

The percentage of mutant SOD1 transgenic mice obtained in relation to the equivalent ATF4 genotype of non-transgenic mice is indicated. Total animals obtained for each genotype is indicated.

### Disease onset analysis

For disease onset determination, we measured three different parameters over time for each experimental animal: (i) rotarod performance, (ii) body weight loss and (iii) disease sign scoring. All this information was analyzed for individual animals to calculate disease onset for each parameter. We started the collection of disease-sign data from 5 to 6 weeks after birth for each mouse. For each parameter a day of onset was defined as follows: We measure motor performance with the rotarod test for each mouse three times per week starting at 60 days as described before using an acceleration protocol measured for total 90 seconds [26]. Disease onset was defined as the day before the animal was not able to perform the task. Body weight measurements were performed three times a week, the same day rotarod was performed, and onset was defined as the day before the animal loses more than 5% of the body weight. To determine disease onset by disease sign observations, we monitored and video recorded the appearance of abnormal limb-clasping, wobbly gait, the first signs of paralysis in one hind-limb, ruffling fur, and hunched posture. We assigned to each visual observation a score 1 when the symptom appears, 3 when it increases or 5 when the phenotype was severe. For this parameter disease onset corresponded to the day when the total score of the animal was more than 10. The end stage of disease was determined as the time when an animal was not able to right itself up within 30 seconds after being placed on its back. For disease onset and animal survival measurements we used four groups of animals: ATF4^+/+^ non transgenic (7 males and 7 females), ATF4^+/+^/SOD1^G86R^ (7 males and 8 females), ATF4^−/−^/SOD1^G86R^ (4 males and 4 females) and ATF4^−/−^ non transgenic (2 males and 2 females). No differences were observed by gender analysis (not shown). Of note, two animals of the ATF4^+/+^/SOD1^G86R^ and the ATF4^−/−^/SOD1^G86R^ groups (one male and one female in each group) did not learn the rotation rod test and were excluded from this specific analysis.

### Tissue analysis

To monitor SOD1 pathogenesis *in vivo*, animals were euthanized and tissue collected for immunohistochemistry and Western blot analysis at the end stage of the disease. 1.5 cm of lumbar spinal cord tissue was collected and divided into two fractions used for Western blot analysis and histology. For immunofluorescence analysis, tissue was then fixed in 4% paraformaldehyde (PFA) in 0.1 M saline phosphate buffer (PBS) for 48 h followed by standard procedures for immunofluorescence in tissue. In brief, spinal tissue was processed on a sucrose gradient (5%, 10% and 30% sucrose in PBS), cryoprotected with Optimal Cutting Temperature compound (Tissue Tek), and fast frozen using liquid nitrogen. Tissue was longitudinally sectioned (5 µm thick slices) using a cryostat microtome (Leica, Nussloch, Germany), as described previously [39]. Sections were immunostained using the following antibodies: anti-NeuN 1∶300 (MAB377, Millipore Bioscience Research Reagents), anti-Olig-2 1∶200 (ab9610, Millipore Bioscience Research Reagents), and anti-GFAP 1∶1000 (N1506, Dako). Tissue sections were analyzed with an Olympus IX71 microscope and images were captured using a QImaging QICAM Fast 1394 camera.

### Western blot analysis of motor cortex and spinal cord protein extracts

After the animal was anesthetized, the brain was removed and the motor cortex was dissected and immediately frozen for further analysis. In addition, lumbar spinal cord tissue was collected in the same animals and homogenized in RIPA buffer (20 mM Tris pH 8.0, 150 mM NaCl, 0.1% SDS, 0.5% DOC, 0.5% triton X-100) or PBS buffer containing 1% Triton X-100 and a protease inhibitor cocktail (Roche, Basel, Switzerland). Tissue was sonicated on ice and then protein concentration was determined by micro-BCA assay (Pierce, Rockford, IL). The equivalent of 30–50 μg of total protein was loaded onto SDS-PAGE minigels. The following antibodies and dilutions were used: anti-Grp78/BiP, anti-Grp58, anti-PDI, 1∶2,000 (Stressgen, San Diego, CA), anti-XBP-1, 1∶2,000 (Biolegend), anti-GFP 1∶1000, anti-ATF4 (Santa Cruz CA), anti-Hsp90 (Santa Cruz, CA), anti-CHOP 1∶2,000 (Santa Cruz, CA); anti-SOD1 1∶3000 (Calbiochem), anti-V5 1∶5000 (Invitrogen), ERO1Lα 1∶2000 (Novus Biologicals) and LC3 1∶3000 (Cell signaling). In general, for Western blotting we analyzed two ATF4^+/+^/SOD1^WT^ (1 male, 1 female), six ATF4^+/+^/SOD1^G86R^ (3 males and 3 females), and four ATF4^−/−^/SOD1^G86R^ (2 males and 2 females) followed by protein expression quantification using image J software.

### RNA extraction and RT-PCR

Total mRNA was prepared from spinal cord tissue previously homogenized in cold PBS followed by Trizol extraction (Invitrogen, Carlsbad, CA). cDNA was synthesized with SuperScript III (Invitrogen, Carlsbad, CA) using random primers p(dN)_6_ (Roche, Basel, Switzerland). Quantitative real-time PCR reactions employing SYBR green fluorescent reagent were performed in an ABI PRISM 7700 system (Applied Biosystems, Foster City, CA). The relative amounts of mRNAs were calculated from the values of comparative threshold cycle by using β-actin as a control. Primer sequences were designed by Primer Express software (Applied Biosystems, Foster City, CA) or obtained from the Primer Bank (http://pga.mgh.harvard.edu/primerbank/index.html). Real time PCR was performed as previously described [26] using the following primers: bim 5′-CGACAGTCTCAGGAGGAACC3′ and 5′CATTTGCAAACACCCTCCTT3′; C*hop/gadd153* 5′-GTCCCTAGCTTGGCTGACAGA-3′ and 5′-TGGAGAGCGAGGGCTTTG-3 *actin* 5′-TACCACCATGTACCCAGGCA-3′ and 5-′ CTCAGGAGGAGCAATGATCTTGAT-3′.

### Statistical Analysis

All data are expressed as mean and SEM. Results were statistically compared using Student's t-test performed for paired groups.

## Results

### Targeting ATF4 on a mutant SOD1 transgenic mouse model

In order to define the possible contribution of ATF4 to ALS pathogenesis, we cross-bred ATF4 deficient mice with SOD1^G86R^ transgenic animals (the equivalent of human SOD1^G85R^) in a C57BL/6J genetic background. This ALS model was chosen since the expression of mutant SOD1 is driven by the endogenous *sod1* promoter, and SOD1^G86R^ encodes an enzyme with minimal SOD1 activity. To generate experimental animals with proper littermate controls, we then crossed ATF4^+/−^ with ATF4^+/−^/SOD1^G86R^ animals and monitored disease progression. Using this breeding strategy, we obtained a total of 463 animals. As predicted, the probability of obtaining a wild-type or a mutant SOD1 transgenic animal in a wild-type background were almost identical (Table 1). In sharp contrast, ATF4 haplo-insufficiency decreased the percentage of SOD1^G86R^ mice generated to 36% (N = 231 ATF4^+/−^ animals versus N = 134 ATF4^+/−^/SOD1^G86R^ mice) (Table 1). As previously described, the generation rate of ATF4 deficient animals was lower than expected with a Mendelian rate calculation, which is possibly due to alterations in amino acid metabolism [11]. Similar results were obtained when the generation rate of ATF4^−/−^ versus ATF4^−/−^/SOD1^G86R^ was analyzed (Table 1). Taken together, these results suggest that ATF4 deficiency reduces the probability of generating experimental mutant SOD1 transgenic mice.

### ATF4 deficiency delays disease onset and prolongs life span in mutant SOD1 mice

**Figure 1:**
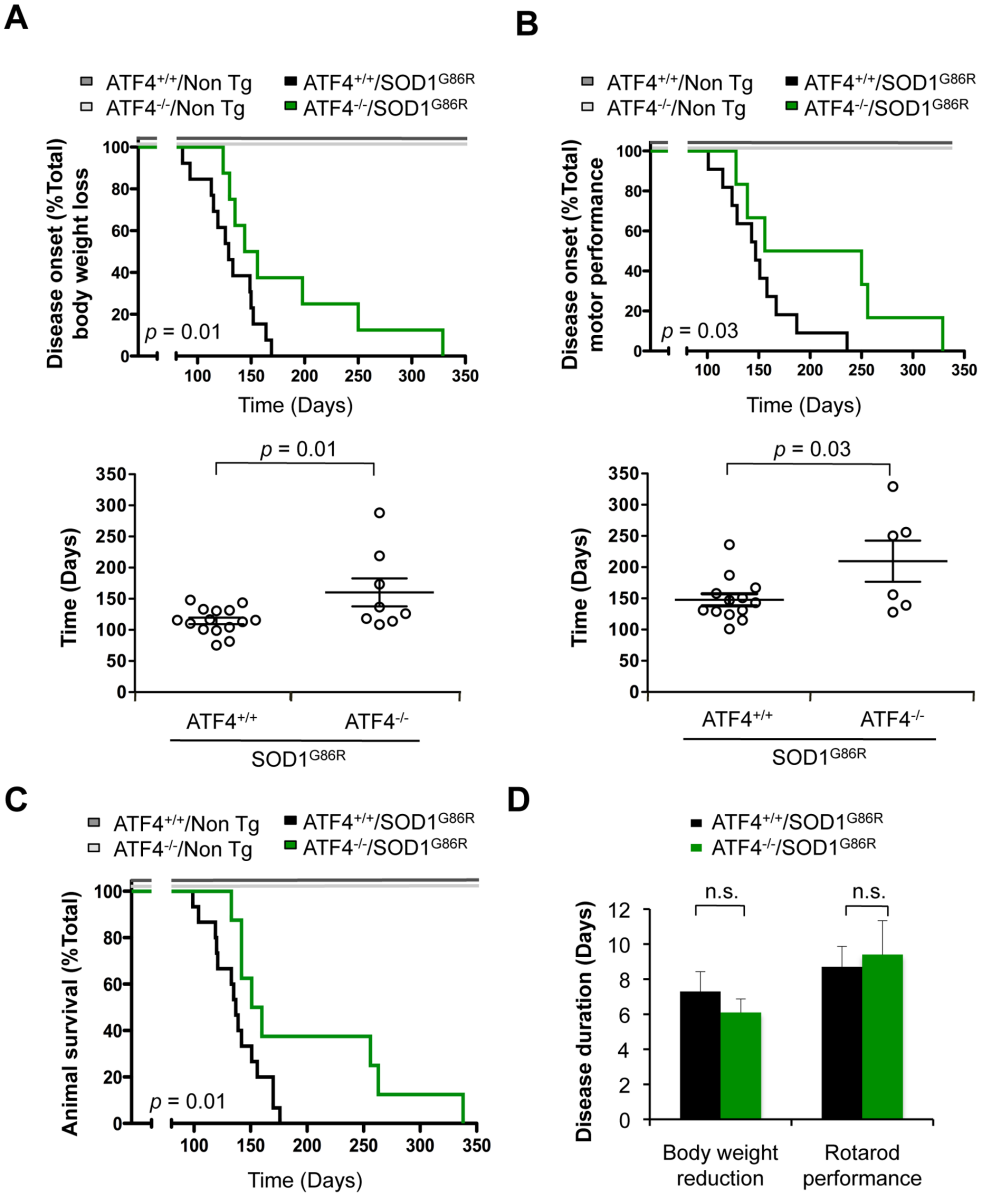
Effects of ATF4 deficiency on disease onset and life span of mutant SOD1 transgenic mice. (**A**) Body weight was monitored in SOD1 transgenic mice (SOD1^G86R^) on an ATF4 wild-type (ATF4^+/+^) or knockout (ATF4^−/−^) background. Disease onset was defined as the day when animals lost 5% of total body weight. Bottom panel: The average of disease onset with this parameter was 130 days for ATF4^+/+^/SOD1^G86R^ (N = 15) and 183 days for ATF4^−/−^/SOD1^G86R^ (N = 8). (**B**) In parallel, rotarod performance was monitored in animals presented in B. Disease onset was determined as the time when animals were not able to perform the task. With this parameter average onset was 147 days for ATF4^+/+^/SOD1^G86R^ mice (N = 13) and 209 days for ATF4^−/−^/SOD1^G86R^ (N = 6). Of note, two animals in each group group were excluded on this analysis because they did not learn the rotarod test. (**C**) Animal survival was monitored in animals described in A. Life span curves showed an average survival of 137 days for ATF4^+/+^/SOD1^G86R^ animals (N = 15) and 155 days for ATF4^−/−^/SOD1^G86R^ (N = 8) mice. In A-C the analysis of control ATF4^+/+^ (N = 14) and ATF4^−/−^ (N = 4) non-transgenic (Non Tg) groups is also presented. (**D**) The duration of the disease was calculated by comparing the measurements of individual animals for life span and disease onset. In all graphs, mean and standard error is presented. *p* values were calculated with Kaplan-Meier statistics and indicated in the Figure. n.s.: non significant.

Taking into account that we were able to generate viable ATF4^−/−^/SOD1^G86R^ mice, we monitored disease progression and survival of these animals. We measured body weight progression and motor performance overtime starting from early-asymptomatic disease stage. Based on a previous report using *perk^+/−^* mice [28], we expected that ATF4 deficiency would exacerbate the progression of experimental ALS. Opposite to our initial prediction, we observed a significant delay in disease onset of ATF4^−/−^/SOD1^G86R^ mice assessed both by the reduction in body weight (Figure 1A) and a decrease in motor performance using the rotarod assay (Figure 1B). Analysis of body weight loss indicated that, the average disease onset obtained was 130 days for the ATF4^+/+^/SOD1^G86R^ group compared with 183 days for ATF4^−/−^/SOD1^G86R^ mice. Determination of disease onset using the rotarod assay also showed a significant delay on disease progression of ATF4 deficient mice, where the average disease onset was 147 days for the ATF4^+/+^/SOD1^G86R^ group compared with 209 days for ATF4^−/−^/SOD1^G86R^ mice. Although we obtained eight ATF4^−/−^/SOD1^G86R^ mice, two of them did not learn the rotarod task and were excluded from this analysis.

Mutant SOD1 transgenic mice showed a consistent survival curve with low variability, with an average life span of 137±5,4 days (Figure 1C). A robust and significant increase in the life span of ATF4^−/−^/SOD1^G86R^ mice was observed (average 155±27,1 days) compared with littermate control ATF4^+/+^/SOD1^G86R^ animals, with an extension in the life span of individual animals ranging from 5 to 101 days longer than the average ATF4^+/+^/SOD1^G86R^ mice (Figure 1C; *p* = 0.01). The duration of the symptomatic phase of the disease, calculated from the time of onset with the two parameters tested to the death of the animal, was similar in ATF4^−/−^/SOD1^G86R^ and SOD1^G86R^ mice on a wild-type background (Figure 1D). Thus, ATF4 deficient animals were more resistant to develop experimental ALS characterized by a significant delay in disease onset and increased life span.

### ATF4 deficiency leads to altered mutant SOD1 aggregation and histopathological features

**Figure 2:**
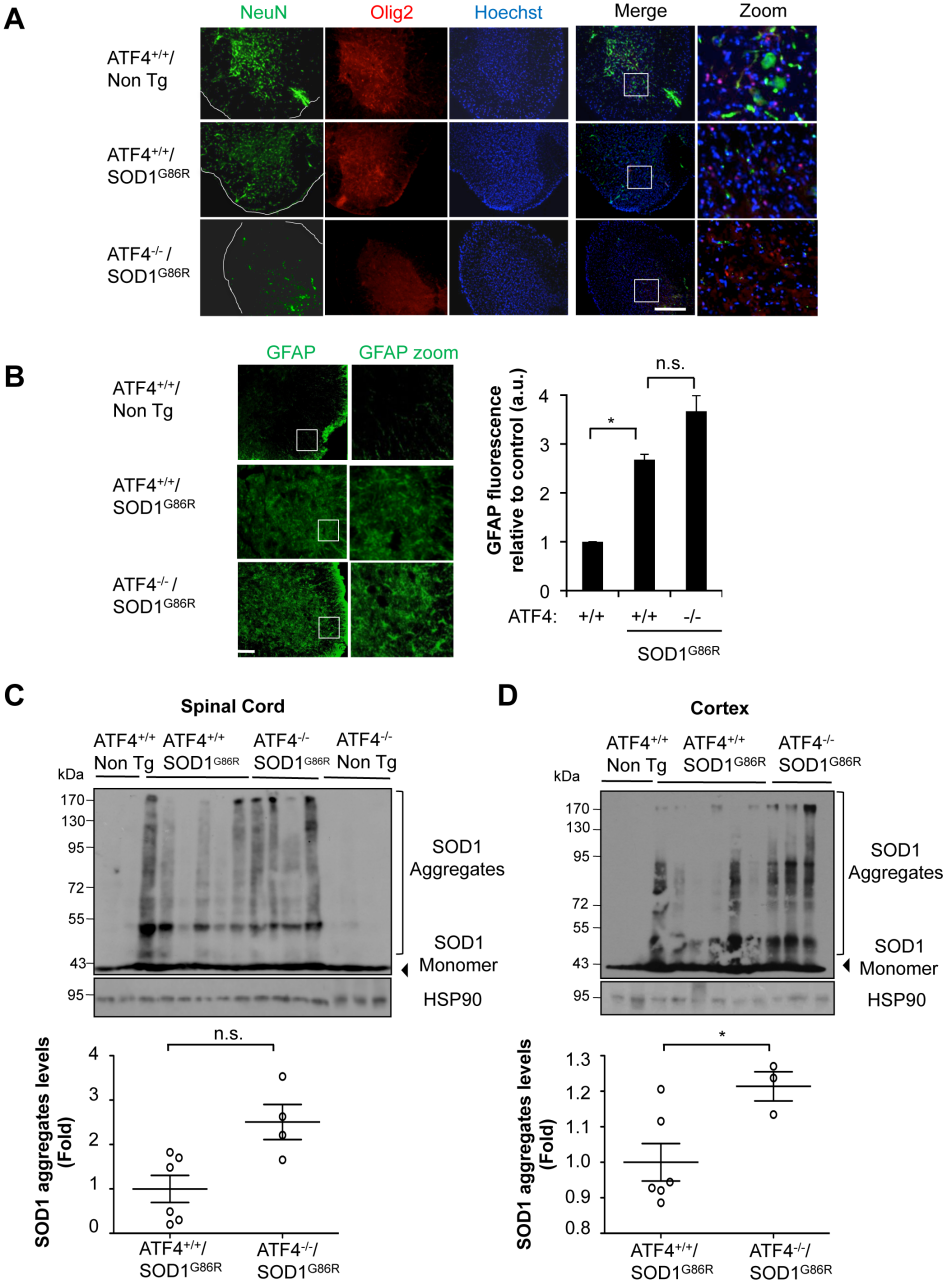
Targeting ATF4 alters SOD1 aggregation in mutant SOD1 transgenic mice. Histological characterization of ATF4^−/−^/SOD1^G86R^ spinal cord. (**A**) Immunofluorescence analysis of NeuN and Olig2 staining in spinal cord tissue derived from ATF4^+/+^, ATF4^+/+^/SOD1^G86R^ and ATF4^−/−^/SOD1^G86R^ at the late disease stage. Hoechst staining is also presented. A merged image of the three staining is provided together with a zoom of the selected area. Bar 250 μm. A representative image is presented of the analysis of three independent animals. (**B**) In the same tissue described in A, GFAP staining was performed and quantified. A zoom of the selected area is also provided (right panel). The border of the spinal cord tissue is delineated with a white line Bar 100 μm. (**C**) SOD1 aggregation was determined in spinal cord protein extracts derived from ATF4^+/+^(N = 2) ATF4^+/+^/SOD1^G86R^ (N = 6) ATF4^−/−^/SOD1^G86R^ (N = 4) and ATF4^−/−^ (N = 3) mice at the symptomatic stage using Western blot analysis. Each well represents an independent animal. (**D**) In parallel, brain cortex was analyzed as described in C. Bottom panels of C and D: Mutant SOD1 aggregation was quantified from experiments presented in C and D, and normalized with the value of HSP90. Average signal of control transgenic mice was normalized to 1. Mean and standard deviation is presented in all plots. *p* values were calculated with Student's t-test, *: *p*<0.05.

We then analyzed ALS-related histological features in the spinal cord of ATF4^−/−^/SOD1^G86R^ and littermate control mice. NeuN staining was performed to visualize neuronal survival in the ventral horn of the spinal cord, showing no significant differences in the neuronal content of ATF4^+/+^/SOD1^G86R^ and ATF4^−/−^/SOD1^G86R^ mice at the end stage of the disease (Figure 2A). The low reactivity to NeuN in SOD1 transgenic mice at this disease stage is due to a massive loss of motoneurons in the model [39]. Similarly, ATF4^−/−^/SOD1^G86R^ mice presented similar content of Olig2-positive cells, which identifies mature and precursor oligodendrocytes, compared to SOD1^G86R^ littermate control animals (Figure 2A). In addition, no differences in GFAP staining were observed in ATF4^−/−^-SOD1^G86R^ mice compared with the ATF4^+/+^-SOD1^G86R^ mice (astrocyte marker, Figure 2B). At basal conditions, all these histological parameters were similar in both ATF4^−/−^ and ATF4^+/+^ mice as we recently reported [39] (Figure S1).

We then monitored the levels of mutant SOD1 aggregation in the frontal brain cortex and spinal cord tissue using total protein extracts in the absence of a reducing agent to visualize mutant SOD1 aggregates. Although variable levels of mutant SOD1 aggregation was observed at the late stage of the disease, an overall tendency to increase SOD1 aggregation was detected in the spinal cord (Figure 2C) and brain cortex (Figure 2D) of ATF4^−/−^/SOD1^G86R^ mice, where only the latter showed significant differences after statistical analysis. The levels of the monomeric form of SOD1 were not affected by ATF4 deficiency.

### Altered expression of ER chaperones and apoptosis-related genes in mutant SOD1 mice deficient for ATF4

**Figure 3:**
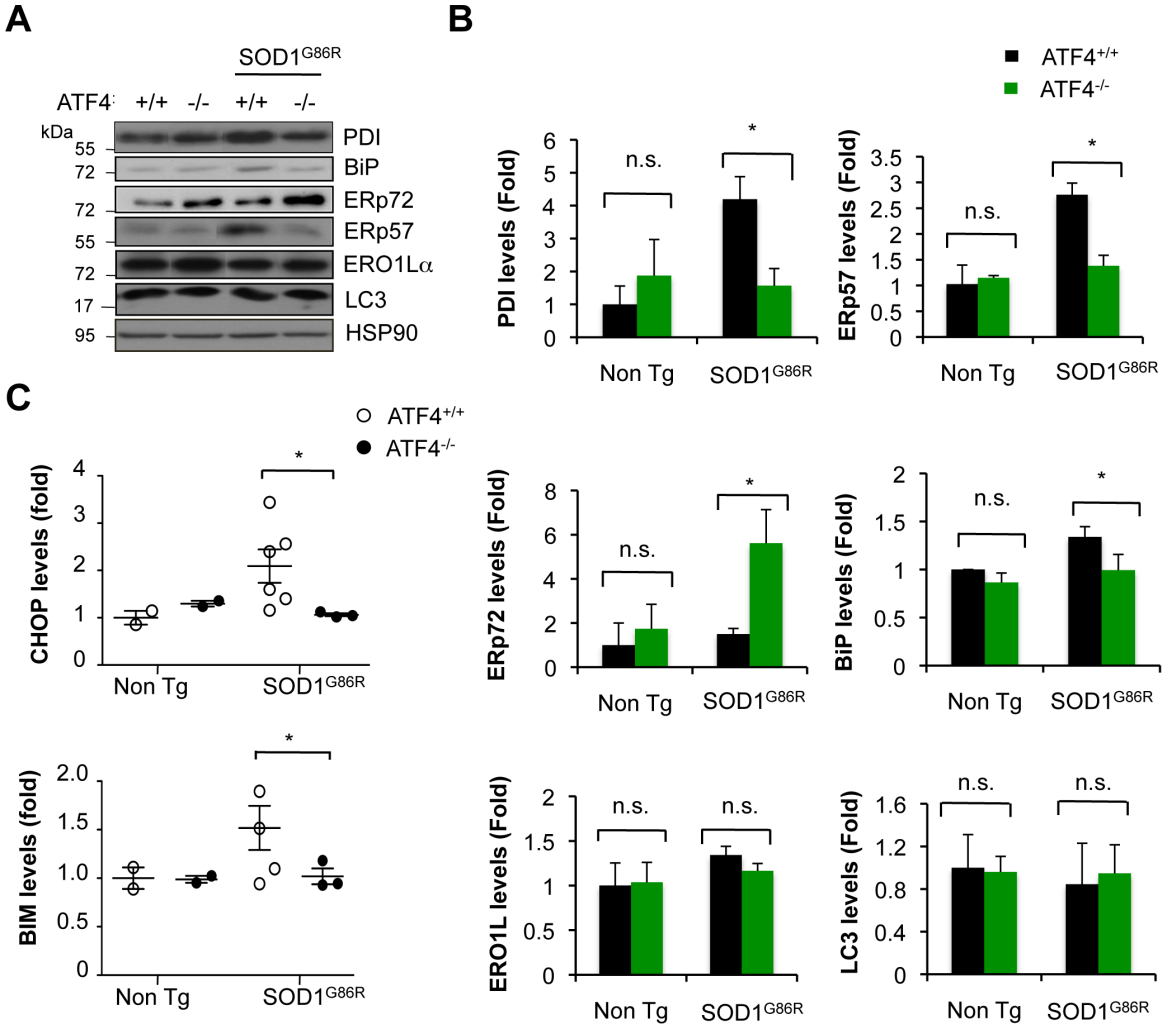
Altered expression of UPR-target genes in mutant SOD1 transgenic mice upon deletion of ATF4. (**A**) Expression levels of several ER stress-responsive proteins were monitored in spinal cord extracts derived from ATF4^+/+^, ATF4^−/−^, ATF4^+/+^/SOD1^G86R^ and ATF4^−/−^/SOD1^G86R^ symptomatic mice using Western blot analysis. The image was composed from representative bands cropped from the same gel and film. (**B**) Quantification of experiments presented in A. ATF4^+/+^ (N = 3), ATF4^−/−^ (N = 3), ATF4^+/+^/SOD1^G86R^ (N = 6) and ATF4^−/−^/SOD1^G86R^ (N = 3). HSP90 was used as loading control, and values were normalized to the average signal obtained in ATF4^+/+^ mice. (**C**) In parallel, CHOP and BIM protein levels were monitored in animals described in A. Mean and standard error is presented. *: *p*<0.05, calculated with Student's t-test. n.s.: non significant.

Protein disulfide isomerases (PDIs) are important UPR target genes that constitute a large family of thiol-disulfide oxidoreductases responsible for the formation of disulfide bonds, playing important roles in folding and protein quality control, in addition to contribute to the redox status of the ER [40]. PDIs levels are induced in ALS-derived tissue, which may have an impact on SOD1 aggregation (reviewed in [41]). Besides, PDI has been also shown to have a pro-apoptotic activity in models of neurodegeneration [42]. Based on this evidence, we monitored the levels of major PDI-family members in the spinal cord of mutant SOD1 transgenic mice, including ERp57 (also known as Grp58), PDI, and ERp72. Consistent with the role of ATF4 as an ER stress transcription factor, the upregulation of ERp57 and PDI was completed inhibited in ATF4^−/−^/SOD1^G86R^ animals when compared to control SOD1^G86R^ mice (Figure 3A, B). Similarly, a partial reduction in BiP levels was detected in ATF4^−/−^/SOD1^G86R^ spinal cord (Figure 3B). Unexpectedly, ATF4 deficiency further enhanced the expression of ERp72 in SOD1^G86R^ mice, which may represent compensatory mechanism due to ERp57 down regulation as previously suggested [43]. In contrast, ERO1Lα levels (redox modulator of PDIs [40]) or XBP1 mRNA splicing was not affected by ATF4 deficiency (Figure 3B and S2). Since targeting ATF4 modulates autophagy in certain experimental systems, we monitored the rate of LC3 conversion. No induction of total LC3 levels or the active LC3-II form was observed in ATF4^−/−^/ SOD1^G86R^ mice (Figure 3A, B). As positive control NSC34 cells treated with lysosomal inhibitors to induce the accumulation of LC3-II (Figure S3).

ATF4 controls important pro-apoptotic genes including CHOP/GADD153 and the BCL2 family member BIM. We monitored the expression of CHOP and BIM in the spinal cord of mutant SOD1 mice using Western blot analysis and real-time PCR. Increased CHOP and BIM levels were detected in spinal cord tissue derived from SOD1^G86R^ mice, which were completely prevented in ATF4^−/−^ deficient animals at the protein (Figure 3C and S4) and mRNA level (not shown). Overall, these data suggest that ATF4 expression has two distinct consequences in ALS pathogenesis: (i) it modulates mutant SOD1 aggregation, and (ii) drives the expression of several ER foldases and ER stress pro-apoptotic factors.

### Knocking down ATF4 in NSC34 motoneuron cells enhances mutant SOD1 aggregation

**Figure 4:**
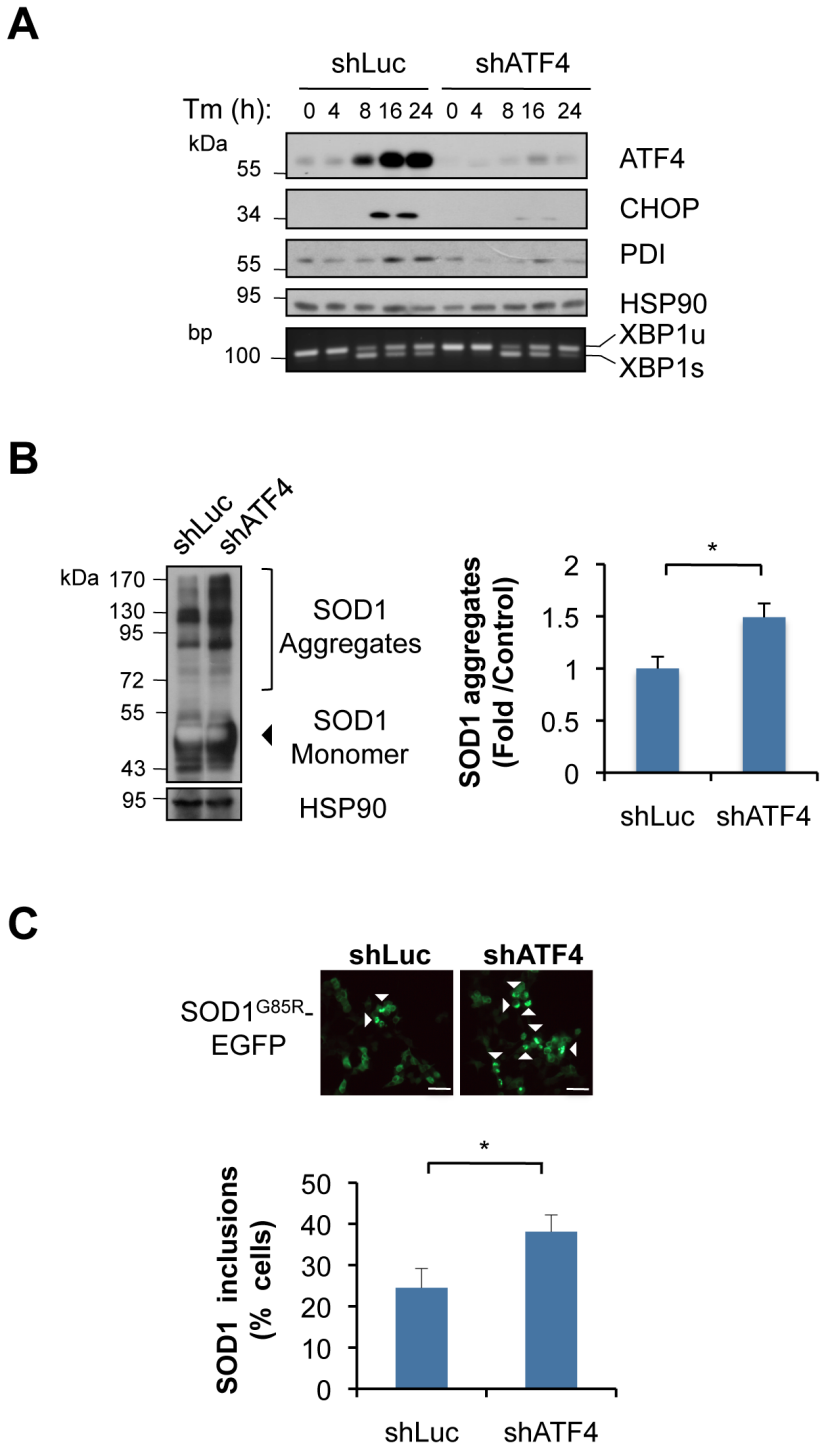
ATF4 deficiency alters the expression of apoptosis genes and enhances mutant SOD1 aggregation in NSC34 motoneuron cells. (**A**) NSC34 cells were stably transduced with lentiviral vectors expressing shRNA against ATF4 mRNA (shATF4) or control luciferase mRNA (shLuc). Expression of ATF4 was analyzed after treatment with 1 μg/mL tunicamycin (Tm) for indicated time. Then, expression of ATF4, CHOP, PDI and HSP90 levels (loading control) was monitored by Western blot analysis. The splicing of XBP1 mRNA is shown by RT-PCR using total cDNA from similar experiments. (**B**) shLuc and shATF4 cells were transiently transfected with expression vectors for SOD1^G85R^-EGFP. After 72 h, mutant SOD1 aggregation was monitored by Western blot. Left panel: quantification of SOD1 aggregation levels normalized with HSP90. Mean and standard deviation is presented from three independent experiments. (**C**) In parallel, mutant SOD1 inclusions were visualized by fluorescent microscopy and quantified (lower panel, bar, 50 μm). A minimum of 150 cells per group was counted. Mean and standard deviation is presented from three independent experiments.

To further investigate the contribution of ATF4 to mutant SOD1 pathogenesis, we knocked down ATF4 in NSC34 motoneuron-like cells. NSC34 cells were stably transduced with lentiviruses expressing a shRNA construct against *atf4* mRNA (shATF4). As control, a shRNA against the luciferase mRNA was employed (shLuc). Knocking down ATF4 was highly efficient as evidenced by a drastic reduction of ATF4, CHOP and PDI expression in cells treated with the ER stress agent tunicamycin (Tm) (Figure 4A). As expected, targeting ATF4 did not affect XBP1 mRNA splicing (Figure 4A). Transient expression of mutant SOD1 in shATF4 cells led to enhance aggregation of mutant SOD1 by 50% as determined by Western blot analysis (Figure 4B). Similarly, an increased number of cells containing mutant SOD1-positive inclusions was observed when human SOD1^G85R^-EGFP was transiently expressed in shATF4 cells (Figure 4C).

### ATF4 regulates the redox state of the cell impacting mutant SOD1 aggregation

**Figure 5:**
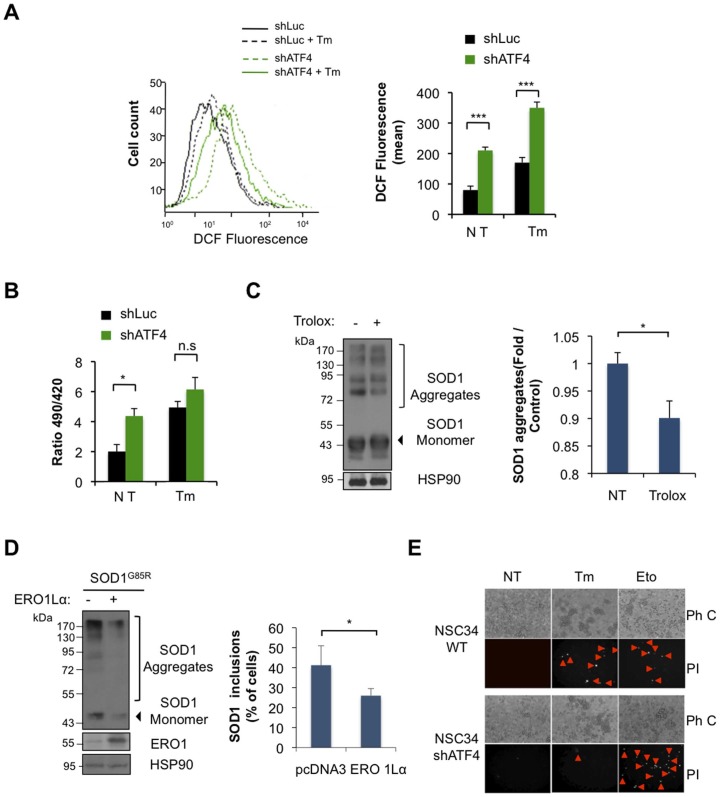
Knocking down ATF4 in NSC34 cells alters the redox state of the ER, contributing to mutant SOD1 aggregation. (**A**) ROS levels were determined in shATF4 and shLuc NSC34 cells at basal levels or after treatment with 1 μg/ml Tm for 16 h using dichlorofluorescein (DCF) staining and FACS analysis. Right panel: Quantification of mean DCF fluorescence. (**B**) The generation of H_2_O_2_ inside the ER was determined in shATF4 and shLuc cells after transient expression of HyPer-ER_lum_ construct. After 72 h, cells were treated or not with 1 μg/ml Tm for 6 h and fluorescence emission determined by live cell-imaging microscopy. (**C**) NSC34 shATF4 cells were transiently transfected with SOD1^G85R^-EGFP plasmid and treated with 400 mM trolox after 24 h. Two days after mutant SOD1 aggregation was analyzed by Western blot. Left panel: quantification of SOD1 aggregates. (**D**) NSC34 shATF4 cells were co-transfected with expression vectors for SOD1^G85R^-EGFP together with ERO1Lα plasmid or empty vector. Left panel: Then, mutant SOD1 aggregation was monitored after 72 h by Western blot (left panel, Bar 50 μm). Right panel: In parallel, mutant SOD1 inclusions were visualized by fluorescent microscopy and quantified. (**H**) Control and shATF4 NSC34 cells were exposed to 5 μg/ml Tm for 16 h and cell death was detected after propidium iodide (PI) staining, and visualized with a fluorescent microscope. As a positive control, cells were treated for 16 h with 20 μm etoposide (Eto). Data is representative of three independent experiments. Arrows heads shows PI stained cells. Ph C: Phase Contrast. In all plots, mean and standard deviation is presented of three independent experiments. *p* value was calculated with Students t-test, *: *p*<0.05.

Based on previous reports linking ATF4 with the regulation of genes related to redox metabolism at the ER [11], we measured ROS content in shATF4 and control cells using dichlorodihydrofluorescein diacetate (DCF) staining, followed by FACS analysis. A dramatic increase in DCF fluorescence was observed in shATF4 cells at resting conditions (Figure 5A), which was further enhanced after induction of ER stress with Tm treatment (Figure 5A). To monitor the redox status of the ER, we expressed the reporter construct HyPerER_lum_ into shATF4 cells, which locally monitors the generation of H_2_O_2_ inside the ER [33]. We measured basal HyPerER_lum_ fluorescence by live-cell imaging microscopy and observed that knocking down ATF4 increased H_2_O_2_ levels in the ER (Figure 5B). Upon ER stress induction these differences were lost (Figure 5B) probably because of saturation of the probe.

We also explored the possible contribution of oxidative stress in the enhancement of mutant SOD1 aggregation found in shATF4 cells. We treated NSC34 cells with the antioxidant Trolox (a derivative of vitamin E), and then transfected cells with expression vectors for SOD1^G86R^-EGFP. After 3 days of expression, a slight, but significant, reduction in mutant SOD1 aggregation was observed when shATF4 cells where treated with Trolox (Figure 5C). To modify the local production of H_2_O_2_ at the ER we overexpressed ERO1Lα [33]. Co-expression of ERO1Lα with SOD1^G86R^-EGFP in shATF4 significantly decreased mutant SOD1 aggregation and the accumulation of SOD1 protein inclusion (Figure 5D). Taken together, these results suggest that knocking down ATF4 in NSC34 cells alters the ER redox environment, influencing mutant SOD1 aggregation. Finally, based on our results in the animal model of ALS and in reports suggesting a protective role of ATF4 deficiency on neuronal survival [21], [22], we analyzed the susceptibility of shATF4 motoneuron cells to ER stress-mediated cell death. As shown in Figure 5E, knocking down ATF4 partially protected cells against Tm toxicity. In contrast, shATF4 cells were sensitive to the toxicity of etoposide (a DNA damage agent). Taken together, these results suggest that ATF4 triggers global changes in ER physiology that alters the susceptibility of cells to protein folding stress.

## Discussion

ALS is part of a large group of neurodegenerative diseases classified as protein conformational disorders that are characterized by the presence of abnormal protein aggregates in the form of inclusions or oligomers in affected neurons [44]. Although a small fraction of ALS cases are linked to mutations in specific genes [1], many studies have shown that the corresponding wild-type proteins are also present in inclusions in human tissue derived from sALS cases, including SOD1 [45], TDP-43 [46], [47], and FUS [48]. Consistent with this concept, the identification of mutations in UBQLN2/Ubiquilin2 [49], p62/SQSTM1 [50], and PDI [51] in ALS cases points out for a critical role of alterations in the proteostasis network in the disease, where the disruption of detoxification mechanisms emerges as a relevant factor for its initiation and evolution. Since ER stress is an early molecular event observed in ALS models, our current study places ATF4 as a relevant modulator of ALS pathogenesis, revealing a new point for future therapeutic intervention.

Our data, together with recent findings, suggests a complex scenario where targeting distinct UPR components in ALS have contrasting effects on disease progression. This notion is revealed by the fact that deleting *xbp1* attenuates ALS in mice [26], whereas *perk* haplo-insufficiency accelerates the pathology. Similarly, pharmacological induction of eIF2α phosphorylation [27], or deletion of ASK1 [29] protects against ALS. Our results demonstrate that the lack of ATF4 has two contrasting effects: it protects against ALS progression in mutant SOD1 transgenic animals, but it also results in a decreased probability to obtain viable animals at birth. The possible cause underlying the decreased generation of ATF4^−/−^/SOD1^G86R^ transgenic mice is unknown. We speculate that the embryonic lethality of the ATF4^−/−^/SOD1^G86R^ may be due in part to a combination of slight developmental defects generated by ATF4 deficiency and the presence of mutant SOD1 transgene. It remains to be determined if targeting ATF4 in adult animals triggers similar protective effects on ALS pathogenesis. It is also interesting to note that, although the SOD1 transgenic line showed a consistent survival curve with little dispersion, ATF4 deficiency generated a large variability in terms of the extension of life span, suggesting differential penetrance of the phenotype observed. Our results suggest that ATF4 expression may alleviate neurotoxic effects of mutant SOD1 occurring even during embryonic development of the nervous system. Then, in adult animals sustained ATF4 expression due to chronic ER stress in ALS may have detrimental effects through the induction of pro-apoptotic signals.

ATF4 has distinct effects in different models of neurodegenerative diseases. For example, we recently reported that targeting ATF4 does not have any consequences on mutant huntingtin aggregation in Huntingtońs disease [52], whereas ATF4 deficiency significantly reduces motor recovery after spinal cord injury [39]. Together with the current study, these reports illustrate the complex function of ATF4 in cell physiology, reflecting the divergent ways of how the UPR may impact specific diseases. ATF4 is a stress-induced protein and a redox sensitive transcription factor that is activated under oxidative stress conditions [11]. Our results agree with the view of ATF4 as a pro-death factor possibly related to its major function as a stress integrator. This is supported by the fact that many metabolic and danger signals converge into eIF2α phosphorylation and ATF4 expression to trigger survival programs or cell death if a critical level of damage has been reached.

Although we detected a slight increase in aggregation of mutant SOD1 in late symptomatic ATF4 deficient animals, these animals showed a delay in ALS progression. We speculate that the strong repression of apoptosis-related genes in ATF4 deficient mice may overcome the pathological events triggered by enhanced SOD1 aggregation observed in these animals. In addition, it may be feasible that that low levels (non-lethal) of oxidative or ER stress in ATF4 deficient neurons may triggers an adaptive response that provides neuroprotection against mutant SOD1 toxicity as suggested in other disease models [53]. Alternatively, it may be also feasible that the generation of large SOD1 aggregates has a neuroprotective role in our experimental system. In fact, it was recently suggested that toxic mutant SOD1 species correspond to a subpopulation of soluble oligomers, where the generation of large SOD1 aggregates may actually provide neuroprotection through the sequestration of toxic species [54]. This hypothesis has also been proposed for Huntington's disease [55] and other protein misfolding disorders.

**Figure 6:**
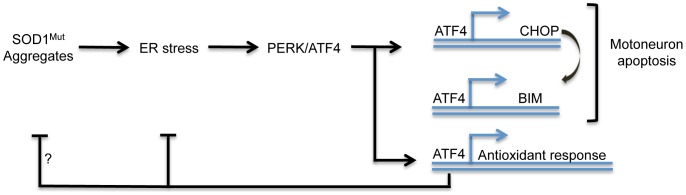
Working model. Accumulation of mutant SOD1 (SOD1^mut^) triggers ER stress engaging the UPR. Expression of ATF4 leads to the expression of genes involved in apoptosis induction including CHOP and BIM, in addition to genes operating in redox control. On a feedback loop, ER stress alters the redox status of the cell enhancing mutant SOD1 aggregation. ATF4 expression may attenuate SOD1 aggregation by controlling the expression of genes involved in redox buffering.

In summary, we postulate that the activation of ER stress by mutant SOD1 induces a chronic response leading to the expression of ATF4-dependent apoptosis genes, in addition to influence the redox status of cell. The latter may feedback on mutant SOD1 enhancing its aggregation (Figure 6). Our results reinforce the concept that ER stress is a key event in ALS pathogenesis, and identifies ATF4 as a novel target for the development of future therapeutic interventions.

## Supporting Information

Figure S1 **Expression of neuronal, oligodendrocite and glial markers in ATF4 deficient animals.** (**A**) Immunofluorescence analysis of NeuN (bar 200 μM), GFAP (bar 100 μM), and Olig2 (bar μM) staining in spinal cord tissue derived from ATF4^+/+^ and ATF4^−/−^ mice.
(TIF)

Figure S2 **XBP1 mRNA splicing in ATF4 deficient animals.** mRNA from motor cortex from ATF4^+/+^ non transgenic animals (ATF4^+/+^ Non-Tg) and deficient for ATF4 (ATF4^−/−^ Non-Tg) was prepared and then cDNA was obtained. The splicing of XBP1 mRNA was measured by RT-PCR followed by PstI digestion which only processes the unspliced form. XBP1 spliced (XBP1s) and unspliced (XBP1u) amplicons are shown. Actin levels were monitored as a loading control. As a positive control, NSC34 cells were treated or not with with 1 µg/ml tunicamicyn (Tm) for 16 h.
(TIF)

Figure S3 **LC3 detection in brain extracts.** Expression levels of autophagy marker LC3 was monitored in spinal cord extracts derived from ATF4^+/+^, ATF4^−/−^, ATF4^+/+^/SOD1^G86R^ and ATF4^−/−^/SOD1^G86R^ symptomatic mice using Western blot analysis. The image was composed from representative bands from the same gel and film. As a positive control for the detection of the LC3-II form, NSC34 cells were treated with a cocktail of lysosomal inhibitors including bafilomycin, pepstatin and E64D (Lys. Inh).
(TIF)

Figure S4 **CHOP and BIM protein expression in mutant SOD1 transgenic mice.** CHOP and BIM protein levels were determined by Western Blot in spinal cord extracts derived from ATF4^+/+^-Non Tg, ATF4^−/−^-Non Tg, ATF4^+/+^/SOD1^G86R^ and ATF4^−/−^/SOD1^G86R^ symptomatic mice. HSP90 was used as loading control. Quantification of these experiments is presented in Figure 3C.
(TIF)
