# Supplementary figures and images for: Functional Contribution of the Transcription Factor ATF4 to the Pathogenesis of Amyotrophic Lateral Sclerosis
Source: PLoS One. 2013 Jul 18;8(7):e66672. doi: 10.1371/journal.pone.0066672 (PMC3715499; doi:10.1371/journal.pone.0066672)

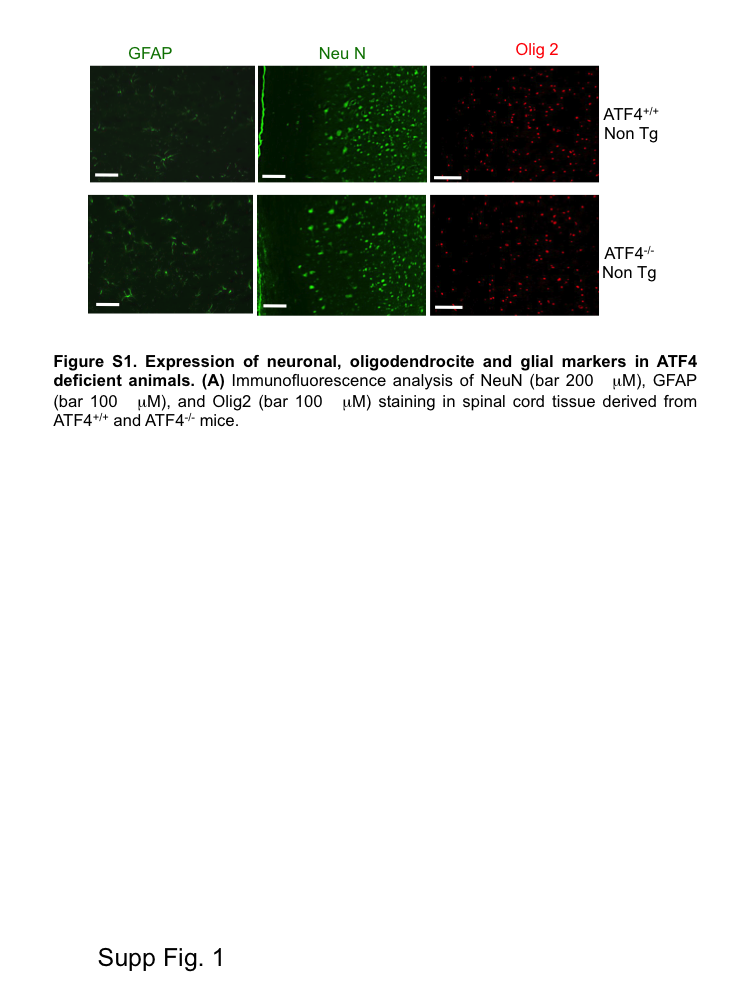

Supplement: Figure S1 — Expression of neuronal, oligodendrocite and glial markers in ATF4 deficient animals. (A) Immunofluorescence analysis of NeuN (bar 200 μM), GFAP (bar 100 μM), and Olig2 (bar μM) staining in spinal cord tissue derived from ATF4+/+ and ATF4−/− mice. (TIF) [file pone.0066672.s001.tif]

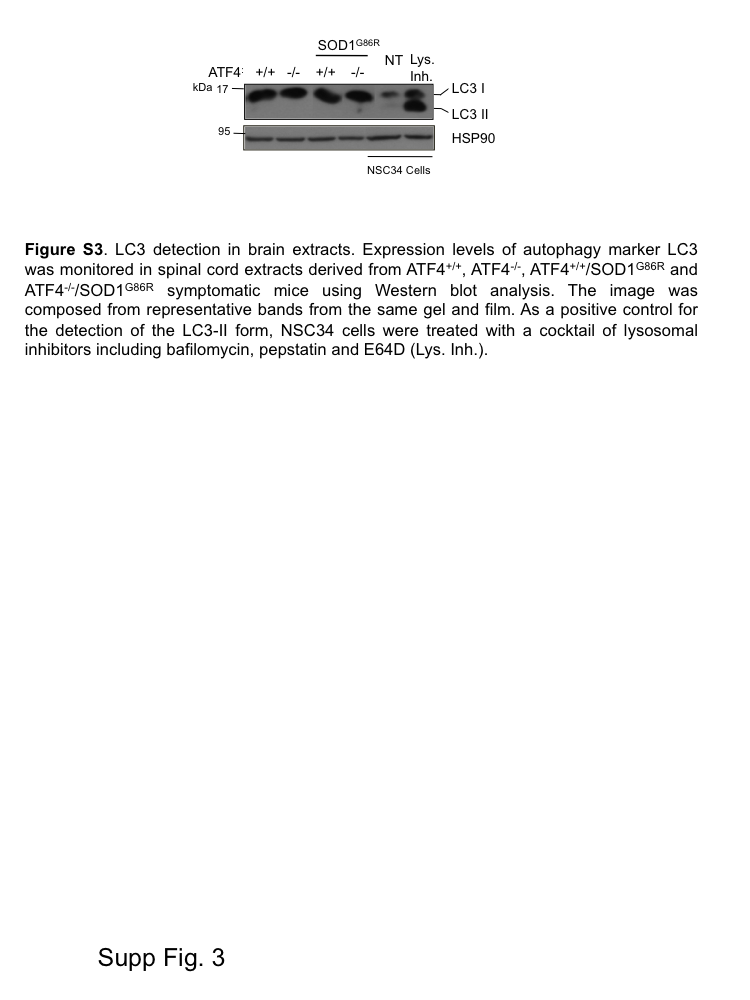

Supplement: Figure S3 — LC3 detection in brain extracts. Expression levels of autophagy marker LC3 was monitored in spinal cord extracts derived from ATF4+/+, ATF4−/−, ATF4+/+/SOD1G86R and ATF4−/−/SOD1G86R symptomatic mice using Western blot analysis. The image was composed from representative bands from the same gel and film. As a positive control for the detection of the LC3-II form, NSC34 cells were treated with a cocktail of lysosomal inhibitors including bafilomycin, pepstatin and E64D (Lys. Inh). (TIF) [file pone.0066672.s003.tif]
